# MLPA-Based Analysis of Copy Number Variation in Plant Populations

**DOI:** 10.3389/fpls.2017.00222

**Published:** 2017-02-21

**Authors:** Anna Samelak-Czajka, Malgorzata Marszalek-Zenczak, Malgorzata Marcinkowska-Swojak, Piotr Kozlowski, Marek Figlerowicz, Agnieszka Zmienko

**Affiliations:** ^1^Institute of Computing Science, Faculty of Computing, Poznan University of TechnologyPoznan, Poland; ^2^Department of Molecular and Systems Biology, Institute of Bioorganic Chemistry, Polish Academy of SciencesPoznan, Poland; ^3^Department of Molecular Genetics, Institute of Bioorganic Chemistry, Polish Academy of SciencesPoznan, Poland

**Keywords:** structural variation, MLPA, 1001 Arabidopsis Genomes project, CNV genotyping, multiplexing

## Abstract

Copy number variants (CNVs) are intraspecies duplications/deletions of large DNA segments (>1 kb). A growing number of reports highlight the functional and evolutionary impact of CNV in plants, increasing the need for appropriate tools that enable locus-specific CNV genotyping on a population scale. Multiplex ligation-dependent probe amplification (MLPA) is considered a gold standard in genotyping CNV in humans. Consequently, numerous commercial MLPA assays for CNV-related human diseases have been created. We routinely genotype complex multiallelic CNVs in human and plant genomes using the modified MLPA procedure based on fully synthesized oligonucleotide probes (90–200 nt), which greatly simplifies the design process and allows for the development of custom assays. Here, we present a step-by-step protocol for gene-specific MLPA probe design, multiplexed assay setup and data analysis in a copy number genotyping experiment in plants. As a case study, we present the results of a custom assay designed to genotype the copy number status of 12 protein coding genes in a population of 80 *Arabidopsis* accessions. The genes were pre-selected based on whole genome sequencing data and are localized in the genomic regions that display different levels of population-scale variation (non-variable, biallelic, or multiallelic, as well as CNVs overlapping whole genes or their fragments). The presented approach is suitable for population-scale validation of the CNV regions inferred from whole genome sequencing data analysis and for focused analysis of selected genes of interest. It can also be very easily adopted for any plant species, following optimization of the template amount and design of the appropriate control probes, according to the general guidelines presented in this paper.

## Introduction

The rise of high-throughput genomics techniques – DNA arrays and, more recently, whole-genome sequencing (WGS) – has revealed the structural complexity and dynamics of eukaryotic genomes. In particular, the ability to re-sequence and compare hundreds or even thousands of genomes of individuals within one species has paved the way for the investigation of the extent to which individual genomes differ from each other. One type of structural variation that is ubiquitous in the genomes of humans, animals and plants is copy number variation (CNV). This term refers to intraspecies duplications and deletions of large DNA segments, usually >1 kb [although variants >50 bp have been recently included in this spectrum (Alkan et al., 2011)]. The human genome is the most intensively studied eukaryotic genome in terms of the distribution and functional significance of CNVs and the mechanisms leading to the formation of copy number rearrangements (Zarrei et al., 2015). However, the number of species for which CNV regions have been inferred on the genome-wide scale is growing rapidly. For plants, this list includes maize, rice, sorghum, *Arabidopsis* (*Arabidopsis thaliana)*, soybean, wheat, and barley (Springer et al., 2009; Beló et al., 2010; Swanson-Wagner et al., 2010; Cao et al., 2011; Saintenac et al., 2011; Zheng et al., 2011; McHale et al., 2012; Muñoz-Amatriaín et al., 2013; Duitama et al., 2015; Bai et al., 2016). As in humans, CNV regions in plants are not uniformly distributed across the chromosomes. Although they are more common in the intergenic regions, they also co-localize with hundreds of protein-coding genes (Swanson-Wagner et al., 2010; Beló et al., 2010; McHale et al., 2012; Muñoz-Amatriaín et al., 2013). The ability to alter the gene structure and copy number makes CNV an important factor that influences gene expression (Żmieńko et al., 2014). By the gene dosage effect, CNVs can also affect the interaction of the genes’ products within protein and metabolic networks (Hanada et al., 2011; Conant et al., 2014). Quite often, such variation accounts for adaptive traits or - as shown for humans - can underlie disease (Stankiewicz and Lupski, 2010; Zarrei et al., 2015). In plants, a growing number of studies highlight the shaping role of CNVs in genome evolution, phenotypic variation and – sometimes rapid - adaptation to environmental challenges (Gaines et al., 2010; Cook et al., 2012; Maron et al., 2013; Chang et al., 2015; Wang et al., 2015). Therefore, it is anticipated that the number of genetic studies focused on individual CNVs of interest will grow and that new CNV-associated traits will be revealed.

In-depth analysis of individual CNVs in plants has rarely been conducted (Gaines et al., 2010; Cook et al., 2012; Maron et al., 2013). Likewise, in plants for which the CNV regions were inferred from WGS data, the subsequent validation was not conducted or was limited to the PCR-based detection of CNV deletions (Swanson-Wagner et al., 2010; Cao et al., 2011; Tan et al., 2012; Bai et al., 2016). Therefore, there is an urgent need to widen the range of experimental studies of CNV in plants to contribute to the creation of high-confidence CNV maps and enhance association studies linking CNVs with phenotypic traits in plant species. In this context, the lack of validated experimental approaches for the analysis of individual CNVs in plants is apparent, as opposed to the well-established methods and standardized protocols available for the human genome.

The range of popular molecular methods used for DNA copy number genotyping in humans is wide (Ceulemans et al., 2012; Cantsilieris et al., 2013; Bharuthram et al., 2014). Among them, multiplex ligation-dependent probe amplification (MLPA), first introduced in 2002 (Schouten et al., 2002) and later developed by the MRC Holland company, is considered a gold standard in the diagnosis of numerous DNA copy number-related human diseases (Hömig-Hölzel and Savola, 2012). MLPA is a simple and robust method of relative quantification of DNA sequences on a population scale. The standard multiplex assay utilizes up to 50 probes targeting specific DNA regions (e.g., exons in a gene of interest). Each probe is composed of two half-probes (physically separate DNA fragments, one fully synthetic and one clone-derived) that match the target sequence in directly adjacent positions with their target-specific sequences (TSSs). Successful hybridization of both half-probes to the genomic DNA enables their ligation and linear amplification. The amplification products are then analyzed by capillary electrophoresis. Relative quantification of the signal peaks from fragments of unique size, generated by individual probes in the assay, provides information about the template DNA copy number. MLPA requires little genomic DNA input (Schouten et al., 2002). Additionally, the genomic sequence targeted by the probes is quite short (50–70 nt), which enables use of MLPA for the analysis of regions too small to be detected by the FISH method. MLPA has been shown to be superior to qPCR for gene copy number quantification (Perne et al., 2009; Cantsilieris et al., 2014). Additionally, it presents similar performance to droplet digital PCR in accurate quantification of up to eight gene copies, making it suitable for the analysis of multiallelic CNVs, i.e., those that exist in more than two genotypes in a population (Zmienko et al., 2016).

According to PubMed, the seminal MLPA work (Schouten et al., 2002) has been cited almost 450 times (∼220 times within 5 last years). Additionally, ∼2,000 articles in PubMed matched the search keyword “Multiplex Ligation-Dependent Probe Amplification”. Among these papers, only 16 also matched the search keyword “plant”. Those that actually described plant applications of MLPA involved alternative applications of this method: the detection of genetically modified organisms (GMO-MLPA) (Rudi et al., 2003), single nucleotide polymorphism (SNP) genotyping (Thumma et al., 2009), or gene expression analysis (RT-MLPA) (Li et al., 2009, 2011, 2013). However, none of these papers presented a primary MLPA application of copy number analysis. Several reasons might account for the fact that the MLPA approach has not been adopted by the plant community. One is much later recognition of the intraspecies variation and CNV prevalence in the plant genomes than in humans. Additionally, the commercial MLPA assays are focused on biomedical studies and cover only humans. Therefore, to assess plant genome variation with MLPA, it is necessary to self-design synthetic probes. It should be noted that, over the years, numerous modifications of the MLPA strategy have been introduced that simplify the probe design procedure (Marcinkowska et al., 2010; Ling et al., 2015, and references therein). In the current work, we present the optimized protocol for MLPA-based CNV analysis and provide guidelines for designing and performing MLPA assays in plants. The protocol is based on the MLPA adaptation developed previously by one of us (PK) that involves fully synthetic oligonucleotide probes, 90 to 200 nt in length, and allows for simultaneous genotyping of >30 different positions in the genomic DNA (Kozlowski et al., 2007). The protocol combines MLPA probe design, synthesis, experimental procedures, data preprocessing and analysis stages into one comprehensive procedure. The lack of MLPA-based genotyping studies in plants highlights the need for such an integrated resource. We also provided the probe design template, developed specifically for the presented MLPA variant. It allows for semi-automatic probe sequence setup, clarifies the idea of probe set composition and shortens the design process by days.

High and low copy level duplications may have different effects on the gene dosage and the phenotype, e.g., by triggering differences in gene expression level or inducing the silencing mechanisms in plants. Therefore, an important aspect of plant CNV genotyping studies is to estimate the actual gene copy numbers in the analyzed lines in order to analyze their influence on the trait of interest (Cook et al., 2014). To illustrate the performance of the MLPA method for precise DNA copy number genotyping in plant populations, we present exemplar assays for 12 genes with different levels of copy number diversity in a population of 80 *Arabidopsis* ecotypes, including multiallelic CNVs. We also describe the set of experimentally verified normalization control probes and the results of genomic DNA template amount optimization performed for this model species.

An advantage of the presented approach is that the assay - after it has been standardized for the particular organism – is always performed in the same conditions, regardless of the probe set composition. It may be utilized for the detailed analysis of a genomic region of interest using a set of MLPA probes scattered along this region or for large-scale validation/genotyping studies of WGS-based predicted CNVs, with 1-2 MLPA probes per inferred CNV.

## Materials and Equipment

### Materials

(1) High-quality genomic DNA for each analyzed sample, evaluated using a NanoDrop 2000 spectrophotometer (Thermo Scientific) and with standard gel electrophoresis; the working concentration is typically 0.4 to 50 ng/μl, depending on the species (see the following sections).
  For *Arabidopsis*: We successfully genotyped CNVs using genomic DNA from 3-week-old rosette leaves extracted with a DNeasy Plant Mini Kit (Qiagen).
(2) Self-designed synthetic oligonucleotides (MLPA half-probes; see the following section for the probe design instructions) purchased from Integrated DNA Technologies (or similar provider) as 100 nmol oligo, purified by HPLC (for oligonucleotides up to 100 nt in length) or PAGE (for oligonucleotides over 100 nt in length); the right half-probes should be additionally modified by 5′ phosphorylation.
(3) Nuclease-free water (not DEPC-treated) (Ambion, cat. no. AM9938)
(4) SALSA MLPA EK-1 reagent kit (MRC-Holland, cat. no. EK1-FAM), which includes the following components:
  SALSA MLPA Buffer
  SALSA Ligase-65
  Ligase Buffer A
  Ligase Buffer B
  SALSA PCR Primer MIX
  SALSA Polymerase
(5) Consumables for capillary electrophoresis, depending on the instrument type; here, for the ABI Prism 3130XL Genetic Analyzer:
  HiDi formamide (Thermo Fisher Scientific, cat. no. 4440753)
  GeneScan 600 LIZ Size Standard (Thermo Fisher Scientific, cat. no 4366589)
  POP7 Polymer (Thermo Fisher Scientific, cat. no 4352759).

### Equipment

(1) 0.2 ml PCR strips and suitable caps, e.g., 8-Strip PCR tubes (Starlab, cat. no. I1402-3500) and 8-Strip caps (Starlab, cat. no. I1400-0800).
(2) Standard and multichannel pipettes.
(3) Thermocycler with heated lid (e.g., Bio-Rad T100 Thermal Cycler or equivalent).
(4) Vortex mixer (e.g., ELMI V-3 Sky Line or equivalent).
(5) Mini laboratory centrifuge with Eppendorf tube adapter and PCR strip adapter (e.g., Labnet Spectrafuge or equivalent).
(6) Capillary electrophoresis instrument (AppliedBiosystems ABI Prism 3130XL Genetic Analyzer or equivalent) or access to a capillary electrophoresis service provider.
(7) Software tool for the extraction of the intensity data after size-separation of MLPA reaction products (e.g., GeneMarker by SoftGenetics).

## Stepwise Procedures

**FIGURE 1:**
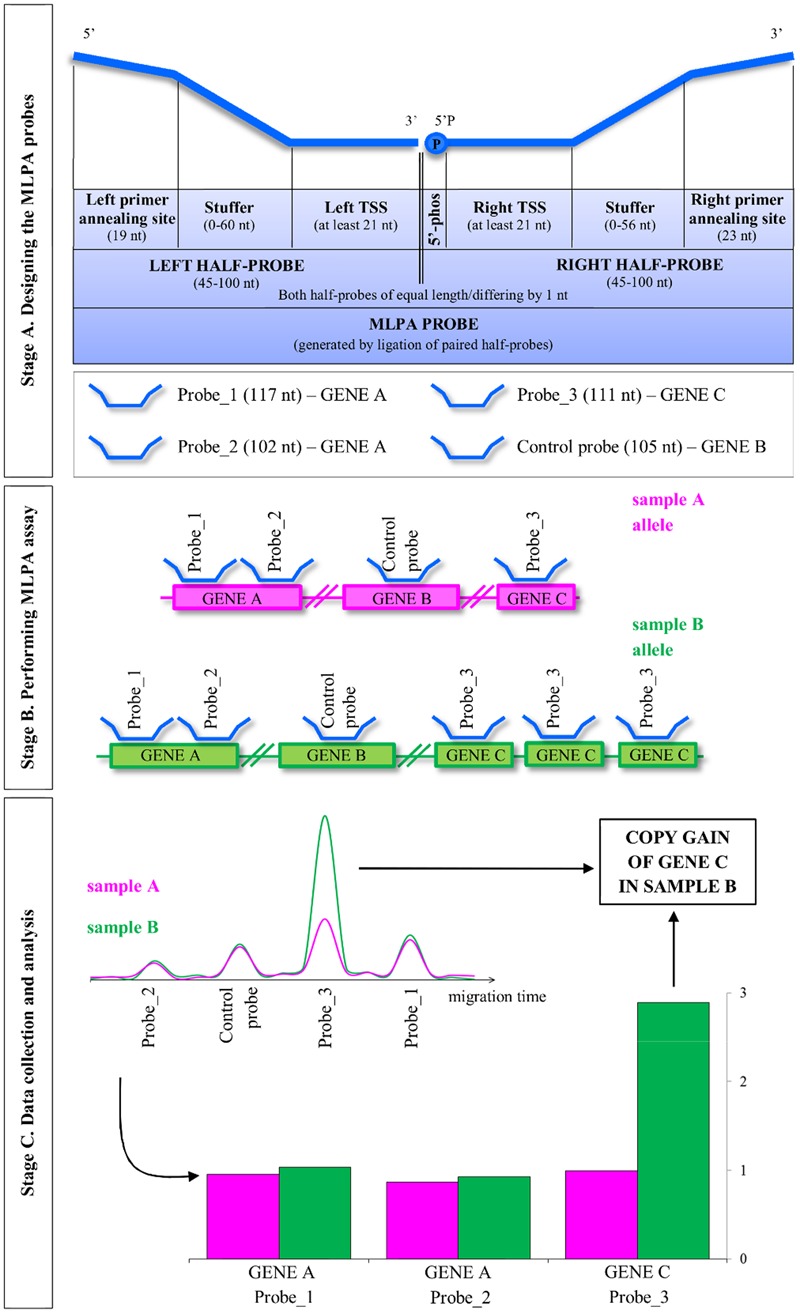
**Overview of the multiplex ligation-dependent probe amplification (MLPA) method.** MLPA is comprised of three main stages: designing the probes, performing the multiplex MLPA assay and data collection and analysis. The three stages are described in the detail in the main text. TSS, target-specific sequence; 5′-phos: phosphorylation at the 5′ end of the oligonucleotide. Note that *Arabidopsis*, as a self-pollinating plant, typically carries pairs of identical alleles. For simplicity, single alleles are depicted.

The general concept of the MLPA strategy is presented in **Figure 1**. The entire procedure involves three main stages: (A) designing the MLPA probes; (B) performing MLPA assay, which involves half-probes hybridization to DNA template, subsequent ligation and amplification; and (C) data collection and analysis, including the estimation of the copy number genotypes.

### Stage A: Design the MLPA Probes (Time: Approximately 1 Week + Oligonucleotide Synthesis and Transportation by an External Provider)

The presented MLPA procedure based on fully synthetic oligonucleotide probes allows for simultaneous copy number analysis of ∼30 individual regions in the genomic DNA. Of these, at least 3 to 5 MLPA probes should target the confirmed non-variable control regions, distant from the studied genomic positions. These probes serve as normalization controls in the subsequent analysis of the MLPA data to account for the possible variation of the input DNA template amount and technical issues. The typical targets of the MLPA assays are protein-coding genes, as the changes in their copy number potentially affect the protein level and may contribute to the phenotype. The number of probes designed for each gene and their density in the covered genomic region depend on the user’s requirements.

The procedure for individual MLPA probe design has been graphically presented in Supplementary Figure S1 and is described in detail in the following sections. We used *Arabidopsis* gene *AT1G01040* encoding Dicer-like 1 protein as an example.

#### Select TSSs for the MLPA Probes

**Step 1.** Retrieve the genomic sequence of the gene of interest from the appropriate database, including the exon-intron positions. We recommend localizing the MLPA probes within the exon sequences because they display lower variation than the non-coding regions of genes.

For *Arabidopsis:* Use the gene locus identifier (e.g., *AT1G01040*) to localize that gene in the TAIR10 genomic sequence, available through the *Arabidopsis* genome browser^1^, and display its splice variants, when applicable (Protein Coding Gene Models track). In *Arabidopsis*, protein coding genes have five exons on average, each with mean length of ∼240 bp (Koralewski and Krutovsky, 2011). This length is sufficient for selecting two adjacent TSSs (one for each half-probe). Use the GBrowse navigation tools to zoom in to the selected exon and export its DNA sequence as a FASTA file.

**Step 2.** Ensure your sequence does not include any repetitive elements.

For *Arabidopsis*, rice, maize, wheat, and some other crops: Submit the extracted sequence to the CENSOR software tool (Kohany et al., 2006) that masks the repetitive elements in the query sequence using the collection of repeats for selected animal and plant species. Select a fragment of at least 100 nt that is not interrupted by any masked regions.

**Step 3.** If possible, check the selected sequence for the presence of SNPs and small indels.

For *Arabidopsis*: Use the 1001 Genomes Project VCF Subset tool^2^ to download the subset of VCF files that contain full-genome VCF data for 1135 accessions (as of September 2016) (1001 Genomes Consortium, 2016). Download SNP information for the region and accessions of interest. Evaluate whether the selected sequence is free of common polymorphisms.

**Step 4.** From the selected region, choose two directly adjacent fragments of at least 21 nt (left and right TSS) and adjust their length and position so that the melting temperature (Tm) of each fragment will be as close as possible to 71°C (calculated with the free RaW program available from MRC Holland^3^ with the following settings: method Go-Oli-Go, salt concentration 0.1 M, oligo concentration 1 μm). Avoid long homopolymer tracts and GC tracts of ≥4 bases.

**Step 5.** Join the adjacent left and right TSSs and use the resulting sequence in a homology search against the genomic sequence of the analyzed species to check for its specificity.

For *Arabidopsis*: Perform a BLAST search against *A. thaliana* NCBI reference genome with the following parameters: blastn algorithm, word size 7, match/mismatch scores 2;-3, gap costs 5;2, no sequence masking and filtering, *E*-value threshold 0.001.

**Step 6.** Repeat steps 3 to 5 until the pair of adjacent TSSs that satisfies all design criteria is found for a given gene.

#### Design the Half-Probes

**Step 7.** Add the respective PCR primer annealing sequence to each TSS and – optionally – the stuffer sequence, in the following order (see **Figure 1**):

for the left half-probe:

5′-left primer annealing sequence – stuffer – left TSS -3′,

where the left primer annealing sequence is GGGTTCCCTAAGGGTTGGA;

for the right half-probe:

5′-right TSS – stuffer – right primer annealing sequence – 3′,

where the right primer annealing sequence is TCTAGATTGGATCTTGCTGGCGC.

For the stuffer, use the fragment of enterobacteria phage M13 sequence (NCBI/GenBank ID V00604, range: 3-119). This fragment has no significant blastn matches to any eukaryotic genomic sequence deposited in the NCBI/RefSeq Representative Genome Database (accessed July 4th, 2016). It has been successfully applied as a stuffer in our previous MLPA assays performed for *Arabidopsis* and human DNA (Marcinkowska-Swojak et al., 2014; Klonowska et al., 2015; Zmienko et al., 2016).

*Note:* The addition of the optional stuffer sequence allows the user to adjust the length of the half-probes so that the resulting PCR amplification fragments would be of unique size and differ by 3 nt for probes in the 90-120 nt range and by 4 nt for probes >120 nt long. The length of the two half-probes in the pair should be the same or differ by 1 nt. For example, to obtain the MLPA probe of length 120, the left and right half-probe sequences should each be 60 nt long (and at least 21 nt of each half-probe should constitute TSS).

To facilitate the process of MLPA probe design and combining multiple MLPA probes in one experimental assay, we provided a Microsoft Excel template (Supplementary Table S1). This template includes the formulas that automatically adjust the length of the stuffer sequence and add the required adapter sequences to both the left and right half-probes. As a result, the final sequence of the MLPA probe of the desired length is returned. The user can choose the MLPA probe length. Typically, when fewer than the maximal number of MLPA probes are included in the assay, we recommend designing shorter probes to minimize the oligonucleotide synthesis costs. Often, the MLPA assays contain two or more probes targeting adjacent genomic regions. We recommend randomization of these probe MLPA lengths to minimize the influence of the possible biases or artifacts. Likewise, we recommend distributing the control probe lengths to cover the entire range of the MLPA probes in the assay.

For *Arabidopsis*: We provide pre-designed sequences for five control MLPA probes (ctrl1–ctrl5) that target genes located on chromosomes 1, 2, 4, and 5. The first gene is *DCL1*, coding for a RNA helicase involved in microRNA processing. The second gene encodes an oxidoreductase belonging to a zinc-binding dehydrogenase family protein. The third non-variable gene is *APG10*, coding for a BBMII isomerase involved in histidine biosynthesis. The fourth gene is *PDF5*, coding for a prefoldin, involved in unfolded protein binding. The fifth gene is *PS2*, coding for a pyrophosphate-specific phosphatase. The lengths of the probes cover the entire range of the MLPA assay (Supplementary Table S1). The regions were selected as not copy-number variable in *Arabidopsis* based on WGS data and were experimentally validated in 189 natural accessions (Zmienko et al., 2016).

#### Order the Oligonucleotide Synthesis

The synthesis of the designed MLPA probes is typically performed by an external service provider, such as Integrated DNA Technologies (IDT).

**Step 8.** Order the synthesis of left and right half-probes, each as separate oligonucleotides, at a 100-nmol scale. All right half-probes must be additionally modified at their 5′ ends (5′ phosphorylation).

*Caution:* 5′ phosphorylation of the right half-probes is essential for a successful ligation step (described below). The oligonucleotides designed for MLPA assays should be of high purity; therefore, we recommend selecting a PAGE or HPLC purification option, depending on the oligonucleotide length and according to the oligonucleotide manufacturer’s recommendations.

**Step 9.** Re-dissolve the lyophilized oligonucleotides upon arrival in deionized water to a concentration of 20 μM. Alternatively, the oligonucleotides can be re-dissolved in 10 mM Tris-HCl, pH 8.2.

**Step 10.** Store the half-probe stocks at –20°C.

### Stage B. Perform MLPA Assay (Time: 2 Days)

*Note:* When performing the MLPA assay, keep all reagents, stock solutions and working solutions on ice. Set up the reactions in PCR tubes or strips (recommended) at room temperature, unless indicated otherwise. Depending on the user’s experience, we recommend running assays for 8–32 samples at once in 1–4 PCR strips.

*Note:* Whenever applicable, prepare the reagent master mixes for all assayed samples with 10% volume surplus to minimize sample-to-sample variation and save pipetting time. Distribute the master mix to eight tubes of a new PCR strip and then transfer the required amount to all PCR strips containing your samples with a multichannel pipette.

*Note:* Perform all incubation steps in a thermocycler, programmed as specified in **Table 1**.

**Table 1:** Programmed thermocycler conditions for multiplex ligation-dependent probe amplification (MLPA) assay.

Program		Action
**Denaturation (Step 5)**		
98°C, 5 min;		Denature samples.
25°C, ∞;		Cool down samples before removing.
Pause		Proceed to Step 6.
**Hybridization (Steps 9-10)**		
95°C, 1 min;		Hybridize half-probes to their genomic targets.
60°C, 16–20 h;		
54°C, ∞;		Adjust the temperature for the next step.
Pause		Proceed to Step 11.
**Ligation (Step 14)**		
54°C, 15 min;		Ligate adjacently hybridized half-probes.
98°C, 5 min;		Inactivate the enzyme.
20°C, ∞;		Cool down samples before removing.
Pause		Proceed to Step 15.
**Amplification (Step 18)**		
35 cycles of:	95°C, 30 s;	Amplify the correctly ligated MLPA probes.
	60°C, 30 s;	
	72°C, 1 min;	
72°C, 20 min;		Perform final extension of PCR products.
4°C, ∞;		Cool down samples before removing.
End		Proceed to Step 19.

*Caution:* Do not vortex the tubes containing Ligase-65 or Salsa Polymerase enzymes. Likewise, do not vortex the master mixes after adding any of these enzymes.

#### Prepare the MLPA Probe Set Mix

The correctly composed assay should include both half-probes (left and right) for each region of interest. Each pair of half-probes should generate a ligation product of unique length in the assay. The concentration of the MLPA probes in the final reaction mixture is very low (see below); therefore, it is convenient to perform a two-step oligonucleotide dilution during the probe set mix preparation as follows.

**Step 1.** Melt all half-probe stocks constituting one assay.

**Step 2.** Dilute each 20 μM stock with water to a 0.2 μM working solution (200 μl).

**Step 3**. Mix 2 μl of each half-probe working solution and fill to 400 μl with water.

The resulting 1 nM MLPA Probe Set Mix will contain all the desired pairs of half-probes in equal concentrations and is directly applicable in the reaction setup.

*Note:* MLPA Probe Set Mix can be stored at –20°C until later use.

#### Hybridize Half-Probes

For each genomic DNA sample, perform the MLPA assay in a separate tube. We recommend running MLPA assays in multiples of 8 in PCR strips with caps.

*Caution:* Replace the strip caps with new ones at each opening during the entire procedure to prevent cross-contamination.

**Step 4.** Aliquot 5 μl of genomic DNA (0.4 to 50 ng/μl) to individual strip tubes to obtain a final template amount of 2–250 ng per assay, depending on the species.

*Note:* We recommend performing template optimization assays for each species.

For *Arabidopsis:* We successfully performed MLPA assays using 2, 5, 10, 15, 30, 60, and 100 ng genomic DNA per assay (see the next section).

**Step 5.** Insert the samples into the thermocycler. Heat for 5 mins at 98°C then let the samples cool to 25°C.

**Step 6.** Remove the samples from the thermocycler and centrifuge.

**Step 7.** Prepare master mix I. Briefly vortex and centrifuge the SALSA MLPA buffer and MLPA Probe Set Mix. Prepare the adequate amount of the master mix I by mixing 1.5 μl of SALSA MLPA buffer and 1.5 μl of 1 nM MLPA Probe Set Mix per sample, with 10% volume surplus. Vortex and centrifuge the tube.

**Step 8.** Add 3 μl of the master mix I to each denatured DNA sample and mix briefly by pipetting. Close the strips with the new caps and centrifuge. The reaction volume in each tube should be 8 μl.

**Step 9.** Put the samples back into the thermocycler and incubate for 1 min at 95°C, then for 16 to 18 h at 60°C.

**Step 10.** Adjust the thermoblock temperature to 54°C before proceeding to the next step.

*Caution:* Do NOT remove the samples from the thermocycler!

#### Ligate the Hybridized Half-Probes

**Step 11.** Prepare master mix II without enzyme. Briefly vortex and centrifuge Ligase Buffer A and Ligase Buffer B. Mix 3 μl of Ligase Buffer A, 3 μl of Ligase Buffer B, and 25 μl of nuclease-free water per sample, with 10% volume surplus. Vortex and centrifuge the tube.

**Step 12.** Centrifuge the tube containing SALSA Ligase-65 enzyme. Add 1 μl of the enzyme per sample with 10% volume surplus to the master mix II. Mix briefly by pipetting. Centrifuge the tube and store on ice until use. Proceed to the next step without delay.

**Step 13.** Without removing the strips from the thermocycler, add 32 μl of master mix II to each sample. Mix by pipetting and close the strips with new caps. The reaction volume in each tube should be 40 μl.

**Step 14.** Incubate the samples for 15 min at 54°C, followed by heat inactivation of the ligase enzyme (5 min at 98°C). Cool the thermoblock to 20°C and remove the samples.

#### Amplify the Ligated MLPA Probes

**Step 15.** Prepare master mix III. Briefly vortex and centrifuge the SALSA PCR primer mix. Mix 2 μl of SALSA PCR primer mix and 7.5 μl of nuclease-free water per sample, with 10% volume surplus. Vortex and centrifuge the tube.

**Step 16.** Centrifuge the tube containing SALSA Polymerase enzyme. Heat the tube in hands for approximately 10 s, then add 0.5 μl of the enzyme per sample with 10% volume surplus to master mix III. Mix briefly by pipetting. Centrifuge the tube and store on ice until use.

**Step 17.** Add 10 μl of master mix III to each sample and mix by pipetting. Close the strips with new caps and replace in the thermocycler. The final reaction volume in each tube should be 50 μl.

**Step 18.** Perform the PCR comprising 35 cycles of: 95°C for 30 s; 60°C for 30 s and 72°C for 1 min, followed by a 20 min final elongation at 72°C. Cool the thermoblock to 4°C.

**Step 19.** Store the samples at 4°C, protected from light, until the product size-separation (1–3 days).

### Stage C. Collect and Analyze the Data (Time: 1 Day for the Data Collection, Variable for the Analysis)

#### Size-Separate the PCR Products by Capillary Electrophoresis

The product separation should be performed under denaturing conditions on any standard capillary DNA analyzer. The specific run parameters must be adjusted according to the recommendations of the instrument manufacturer.

We typically use the services of the local Molecular Biology Techniques facility (at the Department of Biology of Adam Mickiewicz University, Poznan, Poland) and separate the samples in ABI Prism 3130XL Genetic Analyzer (Applied Biosystems), using the following procedure.

**Step 1.** Each MLPA reaction sample is diluted 20× with nuclease-free water, mixed with 9 μl of HiDi formamide (Thermo Fisher Scientific) containing GeneScan 600 LIZ Size Standard (Thermo Fisher Scientific) and denatured.

**Step 2.** Samples are injected at 1.2 kV voltage and separated on ABI Prism 3130XL Genetic Analyzer (Applied Biosystems) at 15 kV, in POP7 separation matrix (Thermo Fisher Scientific).

#### Analyze the Electropherograms

Evaluate the data quality and extract the signal intensity from the electropherograms. Numerous software tools are appropriate for this purpose. Below, we describe the step-by-step analysis performed with GeneMarker (SoftGenetics) (Supplementary Figure S2).

*Note:* The GeneMarker functions used here are accessible in the limited demo version of the software, freely downloadable from the manufacturer’s web site. The details regarding use of these functions are described in the software manual, also available for download.

**Step 3.** Load the electropherogram data to GeneMarker.

**Step 4.** Analyze the raw data files with the MLPA analysis type option and appropriate DNA standard selected (depending on the capillary electrophoresis conditions). Select the size call method and data normalization approach (Supplementary Figure S2A).

*Note:* GeneMarker software provides two normalization options (intra-sample “Internal Control Probe Normalization” and inter-sample “Population Normalization”) that aim to correct for the variation in signal intensity caused by the differences in the lengths of the probes in the multiplex assay. We typically use the intra-sample normalization against our control probes, although at this step it is not critical, because the range of the probe lengths in our assay (96–200 nt) is much smaller than in the case of commercial MLPA assays (130–490 nt).

*Caution:* Use the same parameter settings for all samples. When applying internal control probe normalization, use the same set of control probes for analysis of all samples in the MLPA assay.

*Note:* At the first analysis of a new MLPA assay, run the analysis for a selection of samples using the “NONE” panel selection. This will allow you to manually create the custom MLPA panel later by indicating the peak positions in your pre-processed samples (see Step 5). If the MLPA panel has already been created, select that panel for the final analysis of all your samples.

**Step 5.** Perform this step for the new MLPA assay only. Manually create the probe panel with the Panel Editor (Supplementary Figure S2B). Use the pre-processed set of representative MLPA electropherograms (see Step 4) to locate and insert the alleles at the expected positions. Label the alleles with the MLPA probe names. If you want to use the “Internal Control Probe Normalization” option during the analysis, mark the control probes as 1. Repeat Step 4 to re-run all samples using the newly created panel.

*Note:* In our assays, all peak sizes consistently appeared ∼3 bp shorter than the theoretical length of their attributed MLPA probes. This is not an unexpected result because the migration times of the peak maxima depend on many factors, including the amount of the sample injected, the temperature and the dye used. The capillary electrophoresis systems estimate the relative allele size (using internal standard) and do not necessarily report the true fragment size (McCord, 2003). Therefore, the observed shift is specific to the system and MLPA assay conditions. As long as the peaks are consistently observed at the same positions in all samples under comparison, it does not influence the peak discrimination and subsequent analysis of the MLPA data.

**Step 6.** Evaluate the quality of individual electropherograms in accordance with the peak pattern of the size standard, the electrophoresis baseline, signal sloping and overall signal intensity. Samples that show abnormalities should be excluded from the analysis.

**Step 7.** Configure the report layout and copy the results to MS Excel or similar program for further analysis (Supplementary Figure S2C).

*Note:* The processed data can be reported as the fluorescence intensity (peak height) or the peak area values for each allele. The choice of the output typically does not affect the downstream data analysis and we obtained comparable results with both options. We preferably use the fluorescence intensity data.

#### Estimate the DNA Copy Number

**Step 8.** Use the normalization controls to perform within-sample normalization of all your sample data before comparison.

*For Arabidopsis:* Use at least 3 of the provided control probes (ctrl1–ctrl5) for normalization. Divide each intensity value by the average intensity of the control probes, separately for each sample.

**Step 9.** For each region analyzed, compare the normalized intensity between the samples. Cluster the samples with the similar intensities and infer the copy numbers from analysis of histograms or two-dimensional plots (see next section). Whenever possible, use the (set of) positive and negative control samples with known copy number status to determine the duplication/deletion intensity thresholds (see the next section for exemplar results).

## Anticipated Results

### Exemplar MLPA Assay

Based on the available WGS data from 1001 Arabidopsis Genomes Project (1001 Genomes Consortium, 2016) and our own analysis of a subset of this data including 80 accessions, originally described in (Cao et al., 2011), we selected 12 genes that overlapped CNVs with various levels of structural complexity. Genes *AT1G47670* and *AT1G80830* do not present copy number changes. Genes *AT1G32300* and *AT4G19520* are biallelic; more specifically, they display presence-absence variation. The remaining eight genes are multiallelic and present duplications (*AT4G27080, AT5G09590*, and *AT5G61700*) or duplications and deletions (*AT1G27570, AT1G52950, AT3G21960, AT4G27080*, and *AT5G54710*). Additionally, gene *AT5G09590* overlaps CNV only partially, whereas *AT1G52950, AT5G54710*, and *AT1G27570* are members of multigene families and are localized in the regions of high structural diversity (manifested e.g., by the presence of adjacent or overlapping CNVs, presence of nearby transposable element genes or the presence of clusters of highly similar paralogs). To present the performance of the MLPA approach we set up a multiplex assay Ath.test for these genes (**Table 2**). We evaluated the genes’ copy number status in 80 *Arabidopsis* accessions, characterized in the first stage of 1001 *Arabidopsis* Genomes Project (Cao et al., 2011). All seeds were obtained from The European *Arabidopsis* Stock Centre^4^ and grown as described previously (Zmienko et al., 2016).

**Table 2:** The probe composition and gene targets of Ath.test assay.

Probe name	Probe length	Target genomic site	Locus ID	Predicted CNV status	Source^∗^
ctrl1	96 nt	Chr1:25593..25645	AT1G01040	Non-variable; normalization control	a
ctrl2	111 nt	Chr4:11476533..11476582	AT4G21580	Non-variable; normalization control	a
ctrl3	124 nt	Chr2:15194440..15194490	AT2G36230	Non-variable; normalization control	a
ctrl4	144 nt	Chr5:7847361..7847414	AT5G23290	Non-variable; normalization control	a
ctrl5	172 nt	Chr1:27465468..27465522	AT1G73010	Non-variable; normalization control	a
mlpaA	160 nt	Chr1:17539289..17539343	AT1G47670	Non-variable	b; c
mlpaB1; mlpaB2	90 nt 148 nt	Chr1:30374276..30374321 Chr1:30373647..30373699	AT1G80830	Non-variable	b; c
mlpaC	93 nt	Chr1:11651708..11651754	AT1G32300	Biallelic	b
mlpaD1; mlpaD2	105 nt 114 nt	Chr1:9575624..9575678 Chr1:9577003..9577055	AT1G27570	Multiallelic	b; c
mlpaE1; mlpaE2	136 nt 196 nt	Chr1:19726669..19726721 Chr1:19727385..19727439	AT1G52950	Multiallelic	b; c
mlpaF1; mlpaF2	99 nt 120 nt	Chr3:7737420..7737467 Chr3:7737872..7737929	AT3G21960	Multiallelic	b; c
mlpaG1; mlpaG2	128 nt 164 nt	Chr4:10641616..10641668 Chr4:10644628..10644679	AT4G19520	Biallelic	c
mlpaH	180 nt	Chr4:13592606..13592658	AT4G27080	Multiallelic	b; c
mlpaI	117 nt	Chr4:17705274..17705327	AT4G37685	Multiallelic	b
mlpaJ1; mlpaJ2	108 nt 156 nt	Chr5:2976409..2976464 Chr5:2978013..2978065	AT5G09590	Multiallelic; part of the gene	c
mlpaK1; mlpaK2	188 nt 102 nt	Chr5:22228424..22228479 Chr5:22229438..22229488	AT5G54710	Multiallelic	b; c
mlpaL	132 nt	Chr5:24796111..24796161	AT5G61700	Multiallelic	c

*^∗^The initial information about the gene CNV status comes from the following resources: a, Zmienko et al. (2016); b, Arabidopsis 1001 Genomes Project; c, our unpublished analysis of the WGS data originally presented in Cao et al. (2011).*

### Optimization of the Template Amount

**FIGURE 2:**
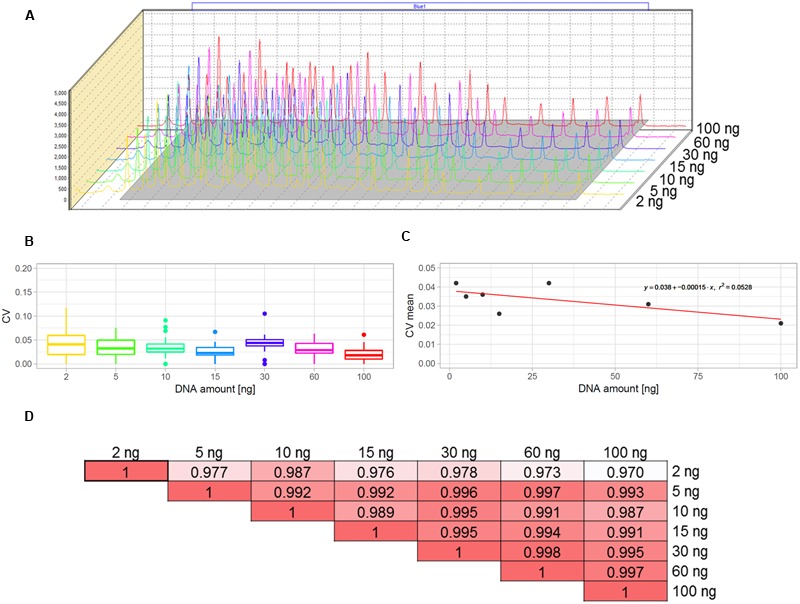
**Optimization of the DNA template amount for copy number variant (CNV) genotyping in *Arabidopsis*.** The data were obtained from three replicates of MLPA assay per each DNA amount tested: 2, 5, 10, 15, 30, 60, and 100 ng. **(A)** comparison of the peak heights and localization on the electropherograms. **(B,C)** Variance of probe intensity measurements across tested DNA concentrations. Boxplots **(B)** present the distribution of coefficients of variation (CV) calculated separately for each probe and each DNA amount; The linear plot **(C)** presents regression analysis (CV vs input amount). For visibility, mean CV values (all probes, each in three replicates) per input amount are displayed on the plot; **(D)** pairwise correlation of results obtained for all template amounts tested, presented as coefficients of determination (*R*^2^) of the MLPA probe intensity data.

The multiplex MLPA-based strategy presented in this paper was originally developed for CNV genotyping of human DNA (Kozlowski et al., 2007; Marcinkowska et al., 2010). To adjust it for use with the *Arabidopsis* genome, we aimed to optimize the amount of DNA template. For humans, the typical MLPA assays include 50-250 ng genomic DNA per reaction. In our previous study, we successfully performed MLPA-based copy number analysis using 100 ng *Arabidopsis* genomic DNA (Zmienko et al., 2016). However, because the *Arabidopsis* genome is ∼20 times smaller than the human genome, we expected that the template amount could be substantially reduced without affecting the reaction performance. To evaluate the acceptable range of DNA amount for this species, we used the Col-0 accession, performed serial dilutions of the DNA template and performed MLPA assays for each of the following DNA amounts: 100, 60, 30, 15, 10, 5, and 2 ng, in three replicates. We observed that the intensity data showed little variance across all DNA concentrations tested and the peaks showed very good resolution and similar distribution, regardless of the template amount (**Figures 2A–C**; Supplementary Data Sheet S1). The normalized signal intensity data for various template amounts were highly correlated, with the results calculated for 2 ng DNA input showing only slightly lowered correlation than the other amounts (**Figure 2D**). From this comparison, we concluded that the whole range of tested DNA amounts generates valid data. Below, we used the smallest tested amount of DNA (2 ng) to perform the exemplar Ath.test MLPA assay.

### Gene Copy Number Analysis

We generated MLPA data, processed it in GeneMarker and exported it to a Microsoft Excel worksheet (Supplementary Data Sheet S1). Three samples were excluded at this stage due to poor data quality. To enable sample-to-sample comparison, we normalized the data within each sample using the mean signal intensity of the control probes ctrl1–ctrl5. The data were then compared and the copy numbers were estimated relative to the Col-0 accession that has the basic copy number of each gene analyzed in this assay (2*n* = 2) and therefore served as the reference sample. To reveal groups of accessions with distinct gene copy numbers, the population data were displayed as dot plots, histograms of the signal intensities or (for genes targeted by two MLPA probes) as 2D plots. We set the duplication/deletion thresholds at <0.7 and >1.3 of the relative intensity, respectively, for all genes in the assay. Subsequently, for each gene, the samples passing the threshold values were clustered and the clusters were manually assigned the copy numbers, as demonstrated previously (Marcinkowska-Swojak et al., 2014; Zmienko et al., 2016).

#### Non-variable Regions

**FIGURE 3:**
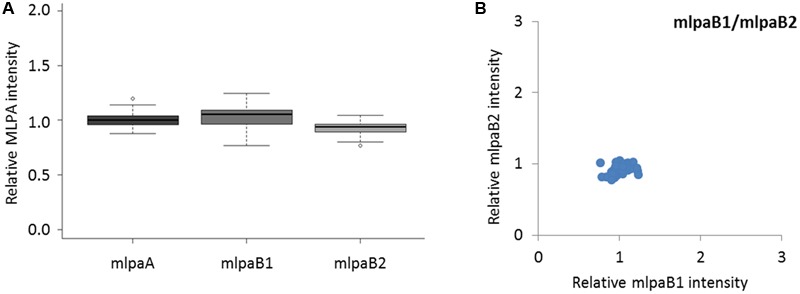
**Multiplex ligation-dependent probe amplification assay results for non-variable regions. (A)** relative intensity distribution of the mlpaA probe, targeting the *AT1G47670* gene as well as mlpaB1 and mlpaB2 probes, both targeting the *AT1G80830* gene, among the accessions; **(B)** 2D plot of intensity from probes mlpaB1 and mlpaB2.

The probes mlpaA, mlpaB1, and mlpaB2 targeted two genes predicted to have the same copy number in all accessions: *AT1G47670*, coding for lysine histidine transporter-like 8 (mlpaA), and *AT1G80830*, coding for NRAMP1 transporter (mlpaB1 and mlpaB2). For all accessions, the relative signals from these three probes were at the same level as those in Col-0 (mean intensity 1.01, 1.03, and 0.93, respectively, see **Figure 3A**) and showed very little variance (CV 0.060, 0.089, and 0.064, respectively). Additional evaluation of the mlpaB1 and mlpaB2 probes on a 2D plot revealed that all samples were grouped in one cluster (**Figure 3B**).

#### Biallelic CNVs

**FIGURE 4:**
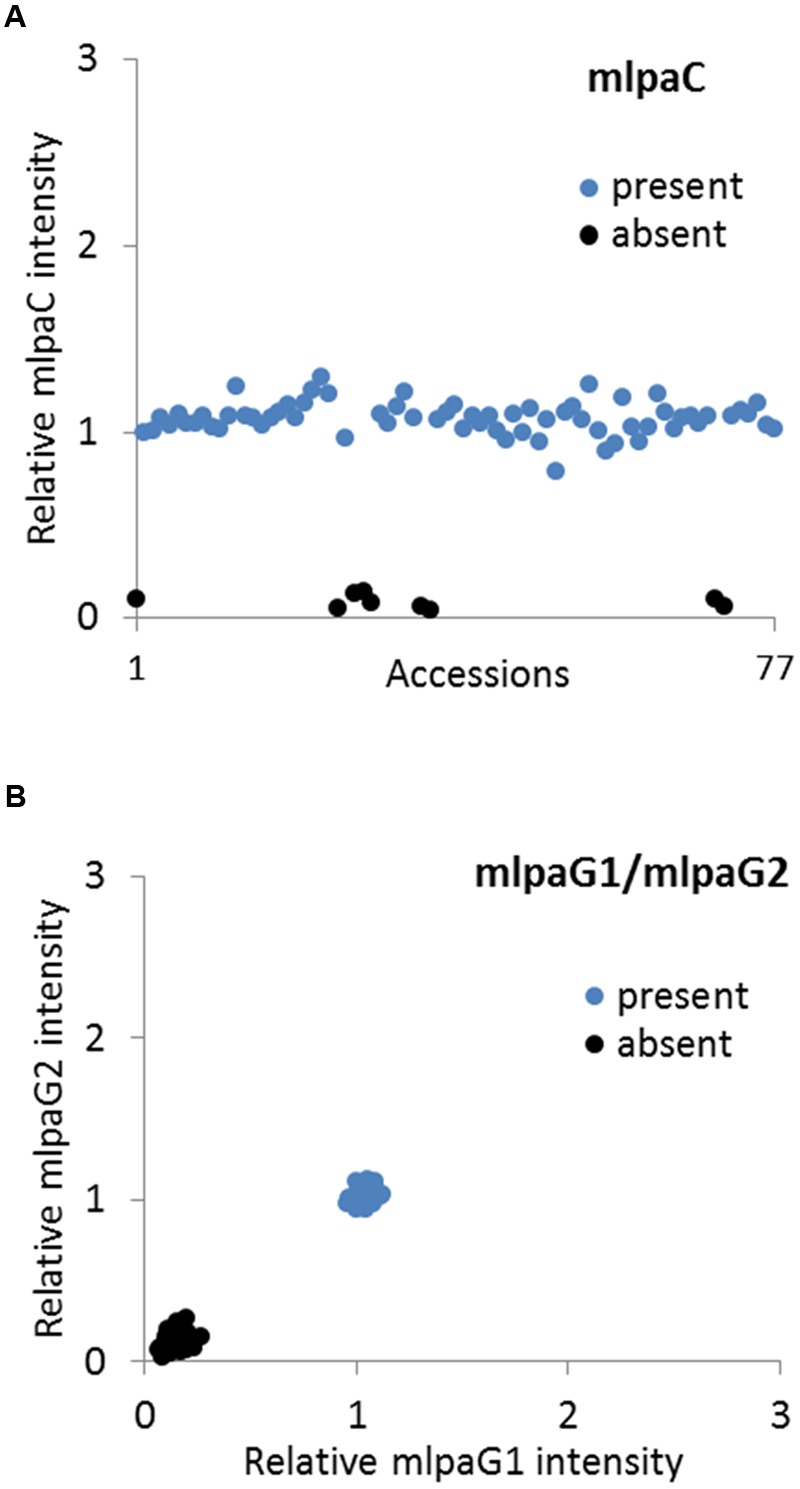
**Multiplex ligation-dependent probe amplification assay results for presence-absence CNVs. (A)** relative intensity from probe mlpaC targeting the *AT1G32300* gene, in individual accessions; **(B)** 2D plot of relative signal from mlpaG1 and mlpaG2, both targeting the *AT4G19520* gene. Clusters are colored according to the deduced CNV status.

We analyzed two genes with presence-absence variation revealed by the WGS data analysis: *AT1G32300* (coding for D-arabinono-1,4-lactone oxidase family protein) and *AT4G19520* (coding for TIR-NBS-LRR class disease resistance protein). We designed one probe (mlpaC) for *AT1G32300* exon 1 and two probes, mlpaG1 and mlpaG2, for *AT4G19520* exons 3 and 5, respectively. For *AT1G32300*, we observed a dominant population of samples with mean signal intensity 1.08, indicative of two gene copies per diploid genome. The remaining samples formed a distinct group with mean signal intensity 0.09, indicative of the absence of the analyzed gene in the respective accessions (**Figure 4A**). In the case of *AT4G19520*, the combined data for the mlpaG1 and mlpaG2 probes revealed the presence of two compact clusters (**Figure 4B**). One cluster included 29 accessions with no difference in copy number relative to Col-0 (mlpaG1 mean intensity 1.03; mlpaG2 mean intensity 1.01). The other cluster included 47 accessions with substantially reduced intensity (mlpaG1 mean intensity 0.14; mlpaG2 mean intensity 0.12), indicative of the deletion.

#### Multiallelic CNVs: One MLPA Probe Per Gene

**FIGURE 5:**
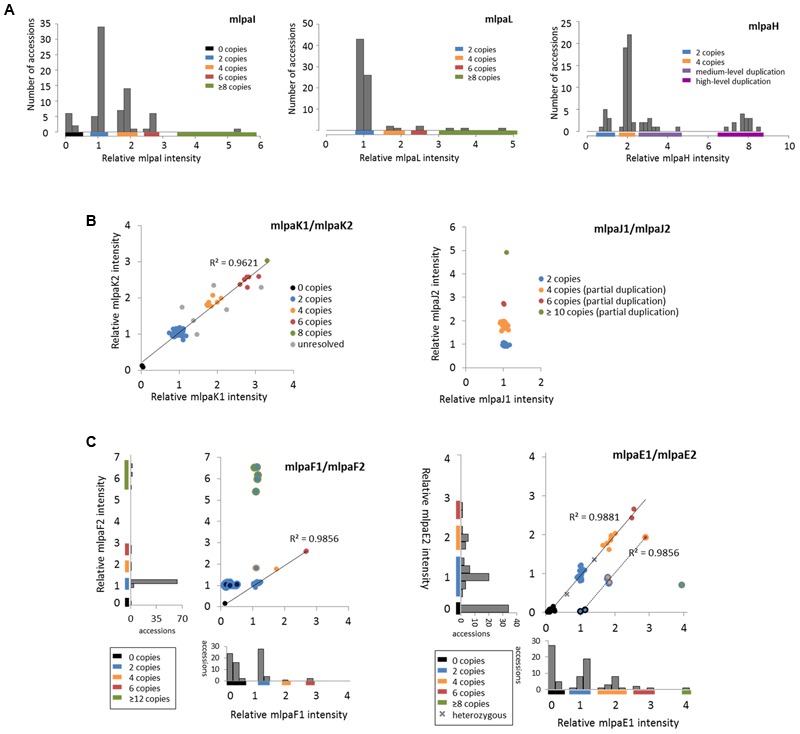
**Multiplex ligation-dependent probe amplification results for multiallelic CNVs. (A)** CNV genotyping with one MLPA probe per gene. Histograms present the relative signal distribution from probe mlpaI (targeting the *AT4G37685* gene), probe mlpaL (targeting the *AT5G61700* gene), and probe mlpaH (targeting the *AT4G27080* gene). The histogram bin size is 0.2 in all plots; **(B)** CNV genotyping with two MLPA probes per gene. 2D plots present the relative signal from probes mlpaK1and mlpaK2 (both targeting the *AT5G54710* gene) and from probes mlpaJ1 and mlpaJ2 (both targeting the *AT5G09590* gene). Clusters are colored according to deduced CNV status. The coefficient of determination (*R*^2^) is calculated for accessions with assigned copy numbers. **(C)** Genotyping complex multiallelic CNVs. 2D intensity plots present relative signal from probes mlpaF1and mlpaF2 (targeting exon 1 and exon 2 of the *AT3G21960* gene, respectively) and from probes mlpaE1and mlpaE2 (targeting exon 6 and exon 9 of the *AT1G52950* gene, respectively). Clusters are colored according to deduced CNV status. The coefficient of determination (*R*^2^) is calculated for subsets of accessions, as detailed in the main text.

For three genes that overlap multiallelic CNVs we designed 1 MLPA probe per gene in Ath.test assay (**Figure 5A**). Gene *AT4G37685* codes for a hypothetical protein and is targeted by the mlpaI probe. Majority of accessions (39) harbor two copies of this gene. Gene deletion was detected in eight accessions and duplication in 30 accessions. Of the latter, 22 accessions had four copies, seven accessions had six copies, and one harbored a very high-level duplication, most likely ≥12 copies.

Gene *AT5G61700* codes for ATH16, a member of ABC transporter subfamily A and is targeted by probe mlpaL. In most analyzed accessions, the gene exists in two copies per diploid genome. In eight accessions, however, duplications were detected: four copies in three accessions, six copies in two accessions, and ≥10 copies in three accessions. It is worth noting that, in MLPA assays, the signal intensity is non-linearly related to the DNA copy number (Zmienko et al., 2016). This is manifested by reducing the distance between the clusters with different duplication levels for high copy numbers. Consequently, a large number of samples harboring high-level duplications is needed to precisely distinguish the clusters of 8 and more copies from each other.

Gene *AT4G27080* codes for a protein disulfide isomerase that is involved in cell redox homeostasis and is targeted by the mlpaH probe. From the WGS data, we predicted that majority of accessions harbor partial or full duplications of this gene. Likewise, MLPA analysis revealed that only nine accessions harbor two copies of *AT4G27080* gene, while duplications were detected in 68 accessions. Among them, we clearly identified a group of 44 accessions with four copies, but the remaining accessions were less distinctive and formed two heterogeneous groups which we named “medium-level duplications” (10 accessions) and “high-level duplications” (14 accessions). For 12 of these “high-level duplication” accessions, the mlpaH peak intensity counts reached the upper detection limits (see **Notes** section below for additional comments). We concluded that designing two or more MLPA probes targeting this genomic region and repeating the assay with adjusted capillary electrophoresis parameters would be helpful in more accurate distinction of the CNV genotypes or resolution of the structural complexity of the investigated gene.

#### Multiallelic CNVs: Two MLPA Probes Per Gene

For 2 other genes that overlap multiallelic CNVs we designed two MLPA probes per gene (**Figure 5B**). The *AT5G54710* gene codes for an ankyrin repeat family protein and is positioned between two other ankyrin repeat family protein coding genes, in the region that is highly copy number variable. We used two specific probes (mlpaK1 and mlpaK2), located in the fourth and third exons of *AT5G54710*, respectively, and confirmed that this gene is multiallelic. The high linear correlation of the mlpaK1 and mlpaK2 probe intensities allowed us distinguish several clusters of accessions with distinct copy numbers: 0 copies (2 accessions), 2 copies (54 accessions), 4 copies (8 accessions), 6 copies (6 accessions), and 8 copies (1 accession). We did not assign the integer copy numbers for 6 accessions which displayed uneven duplication level based on the mlpaK1 and mlpaK2 probe signal.

The *AT5G09590* gene, encoding mitochondrial heat shock protein MTHSC70-2, is localized in the breakpoint of a large CNV that encompasses loci *AT5G09590 – AT5G09630*. Consequently, *AT5G09590* is only partially duplicated in several accessions. We designed two probes, localized outside of and within the CNV region (mlpaJ1, targeting fourth exon and mlpaJ2, targeting sixth exon, respectively). The results of the MLPA assay clearly revealed that only the 3′ part of *AT5G09590* (targeted by probe mlpaJ2) is duplicated: 43 accessions harbored four copies, two accessions harbored six copies, and one accession harbored at least 10 copies. The region targeted by probe mlpaJ1 invariantly had two copies in all accessions.

#### Complex Multiallelic CNVs

Some genomic regions, e.g., these that harbor clustered multigene families, may display high structural diversity in the populations. A gene may be fully duplicated/deleted in some accessions while in the other ones only part of this gene may display copy number alteration. Additionally, the duplicated DNA copies within one sample may differ from each other in length and sequence, which may affect the affinity of the MLPA probe to some (but not all) copies. Consequently, the copy number pattern revealed by the MLPA analysis may be complex. Below we present some examples of MLPA analysis in multiallelic CNVs with a complex structure (**Figure 5C**).

The *AT3G21960* gene is localized in the central part of a ∼50 kb CNV, that encompasses 21 genes, mainly members of the receptor-like protein kinase-related family and genes coding for proteins with unknown domain DUF26. We assayed the *AT3G21960* gene with specific probes targeting exons 1 and 2 (probes mlpaF1 and mlpaF2, respectively). In 30 samples the signals from these probes were highly correlated and formed 4 distinct groups of: 0 copies (1 accession), 2 copies (26 accessions), 4 copies (1 accession) and 6 copies (2 accessions). In 6 accessions, however, only the mlpaF2 probe intensity was elevated (1.83–6.54), while mlpaF1 intensity was about 1. On the contrary, the remaining 41 accessions formed a compact cluster, with the mlpaF1 intensity below 0.7 (the value that has been set as the deletion threshold), and the mlpaF2 intensity about 1. A brief evaluation of the *AT3G21960* genomic sequence inferred from WGS data^5^ (obtained with Pseudogenomes Download Tool) provided evidence that this complex pattern is true, as 519 out of 1135 accessions with available genomic data had 80–100% uncalled sites (Ns) in the exon 1 sequence, while only 3 accessions had 80–100% uncalled sites in exon 2 sequence.

Complex multiallelic CNVs are often related to the activity of mobile genetic elements, which may trigger partial or full deletion/duplication of the nearby genes. Gene *AT1G52950* codes for a nucleic acid-binding OB fold-like protein and is localized within one CNV region with a nearby transposable element gene *AT1G52960* (the two loci are separated by only 3.6 kb distance). We assayed the copy number status of *AT1G52950* using two probes, mlpaE1 to target exon 6 and mlpaE2 target exon 9. For 69 accessions, we detected compact clusters with distinct copy numbers (0 to 6 copies) and a high correlation between the two measurements (*R*^2^ = 0.9881). Interestingly, in two cases, the intensity data suggested the existence of one copy and three copies of the *AT1G52950* gene per diploid genome in the surveyed individuals. *Arabidopsis* is a highly self-pollinating species for which most genomic loci are expected to exist in a homozygous state, therefore assaying additional individuals would be necessary to establish the representative gene copy number for these two accessions in a population study. For seven accessions, of which six originated from Southern Tyrol region and 1 was a Spanish relict accession (1001 Genomes Consortium, 2016), the copy number status indicated by probe mlpaE1 was always higher than the copy number status indicated by probe mlpaE2. This effect may have many reasons, e.g., partial duplication or deletion of a gene of interest, sequence divergence in some duplicated copies that affect the hybridization of one MLPA probe, etc. Unambiguous interpretation of these data would require additional region characterization by sequencing. Nevertheless, the signals from both probes were also well correlated (*R*^2^ = 0.9856). Finally, one accession displayed an extremely high level of duplications at the mlpaE1 target site while no copy number changes were observed at the mlpaE2 site.

#### Effect of Non-specific Hybridization on MLPA Signal

**FIGURE 6:**
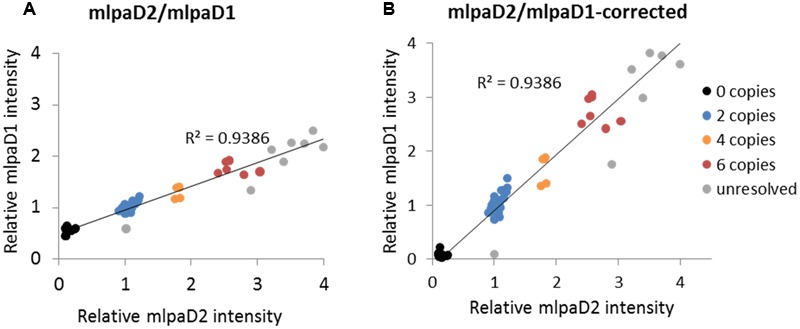
**Effect of non-specific probe hybridization on the MLPA results.** Probes mlpaD1 and mlpaD2 target the *AT1G27570* gene. Additionally, probe mlpaD1 targets also the *AT1G27590* gene. **(A)** 2D intensity plot of relative signal from probes mlpaD1 and mlpaD2; **(B)** 2D intensity plot of relative signal from probe mlpaD1 corrected for the presence of non-specific hybridization signal (see main text for details) and probe mlpaD2. Clusters are colored according to the deduced CNV status and are identical for each sample on both plots. The coefficient of determination (*R*^2^) is calculated for all accessions. The deletion of *AT1G27570* gene has been confirmed by PCR in accessions from the “0 copies” cluster (not shown).

To present the effect of compromised probe specificity on the MLPA results, we assayed a gene *AT1G27570*, which encodes the phosphatidylinositol 3- and 4-kinase family protein and is localized within the large multiallelic CNV (over 20 kb). We designed two probes, mlpaD1 and mlpaD2, targeting this gene, of which only mlpaD2 was specific to *AT1G27570*. Probe mlpaD1 had an alternative target site (with only two mismatches in the left TSS and one mismatch in the right TSS, distant from the ligation site) in the nearby gene *AT1G27590*, not copy number variable. As a result, the signal from the mlpaD1 probe was elevated by the background signal from the alternative target site. This background signal was stable (due to unchanged copy number of *AT1G27590* gene in all accessions) therefore the high correlation between the data for mlpaD1 and mlpaD2 probes was preserved (**Figure 6A**). As a rule, we suggest re-designing of the MLPA probes that produce non-specific signal. However, if a set of the control samples that carry confirmed deletion of the gene of interest can be defined, these samples may be used for the data correction. In the present example, we calculated the mean non-specific signal of probe mlpaD1 in the cluster of 15 samples with gene deletions (marked in black color in **Figure 6A**). This value was then subtracted from the probe mlpaD1 signal in each sample, before estimating the intensity ratio relative to Col-0 accession. The correction improved the relative intensity ratio observed for probe mlpaD1 (**Figure 6B**). We note here, that the process of data correction had no effect on the overall correlation between the signals from probes mlpaD1 and mlpaD2. This correlation was high (*R*^2^ = 0.9386), therefore allowing to distinguish the copy number clusters on 2D plots pretty easily both before and after data correction.

## Notes

Below we included some notes on the limitations of the procedure, common mistakes and possible artifacts related to the presented application.

### Probe Design

Oligonucleotide MLPA probes described in this procedure target specific sequences in the genome, typically 45–75 bp. Regions located outside of the probe’s recognition sequence may have different copy number status. If partial gene duplication/deletion or insertion of duplicated sequence is suspected, additional probes, e.g., covering different exons of the gene should be included in the assay.

Compromised ability of MLPA probe to recognize the target sequence may be the source of false positive results. Sequence changes (SNPs, indels, point mutations) in the target sequence detected by a probe can negatively affect or completely prevent probe binding. The critical positions in the TSS sequence are these constituting the ligation site; the presence of a SNP at or near the ligation site will disrupt the ligation step and result in no signal from the MLPA probe, falsely indicative of deletion of the region in the affected sample (Kim et al., 2016). Note that the MLPA technique can be also used for detecting small mutations (Marcinkowska-Swojak et al., 2016), but these applications are not covered in the present protocol.

The accuracy of the results is also strictly dependent on the MLPA probe specificity. If alternative target site exists in the genome (e.g., in a paralogue or a pseudogene), it will generate non-specific signal (see Effect of Non-specific Probe Hybridization on the MLPA Results Section). To this end, for plants with incomplete genome information we strongly advise designing ≥2 MLPA probes per gene, to minimize this risk.

In the case of newly designed MLPA probes we recommend verifying their performance on a (set of) well characterized reference samples. If no product is observed, make sure that the common mistakes interfering with the experimental steps are avoided (see below). If needed, re-design the MLPA probe.

### Assay Design and Performing

Multiplex ligation-dependent probe amplification results may be compromised by multiple factors that will affect the enzymatic reactions and result in reduced peak signals. These factors include but are not limited to: DNA integrity and contamination, presence of PCR inhibitors in the samples, incomplete DNA denaturation, sample evaporation, suboptimal amount of the sample DNA used. In the Section “Stepwise Procedures” we included useful tips regarding the sample preparation and assay setup. Additional comments are given below.

If the DNA sample contamination is a suspected problem, perform new DNA extraction. From our experience, we advise using column-based methods, e.g., DNeasy Plant Mini Kit (Qiagen) for DNA extraction (or purification of DNA extracted with other methods) because they produce samples of high purity and comparable amounts.

Use multichannel pipettes to reduce the pipetting time and avoid sample evaporation.

Reduce sample-to-sample variability by simultaneous performing multiple assays, using strips (preferable) or multiwell PCR plates. Use the same MLPA Probe Set Mix preparation for all samples under comparison.

Replacing the strip caps on each opening minimizes the risk of sample cross-contamination.

Follow the capillary electrophoresis protocols (size standard, sample preparation, injection time and voltage) suitable for the instrument used. Decrease injection time if the peaks are out of range. We recommend prior optimization of the DNA template amount in the assay and capillary electrophoresis conditions on a validated reference sample.

Abnormal pictures after capillary electrophoresis may indicate capillary electrophoresis problems but they also may result from the PCR step troubles. See the MLPA troubleshooting wizard by MRC Holland^6^ for common peak pattern problems and possible solutions.

### Data Analysis and Copy Number Estimation

It is advisable to manually check the peaks identified by GeneMarker before further data processing. In our assay, we repeatedly observed that the software did not detect the peaks for probe mlpaH in 12 samples and reported “0” intensity for this probe (Supplementary Figure S3). In fact, high intensity peaks from probe mlpaH with their tops flattened (cut) were present in these samples, which indicated that the signal exceeded the capillary electrophoresis system detection limits. We manually corrected the peak localization and used the maximum reported values for copy number calculation, but this likely resulted in underestimation of the gene copy number in these samples in our study (see Section “Multiallelic CNVs: One MLPA Probe Per Gene”). To accurately quantify the probe signal, repeating the electrophoresis with lower injection time would be necessary. The results from high and low injection time electropherograms may be then merged after internal control probe normalization step, to preserve good resolution of the low intensity peaks.

Multiplex ligation-dependent probe amplification is a relative technique, therefore selecting well validated reference samples with basic copy number of the region of interest (usually two copies) is essential for accurate quantification. However, in case of population scale CNV genotyping of numerous independent genomic regions in a multiplex assay (similar to example provided in this paper) such a reference sample may not exist or remains unknown. Providing that sufficiently large number of samples in the population are genotyped, the presented protocol still allows for inferring the cluster copy numbers without a reference sample, under the assumption that the neighboring clusters of accessions/lines differ by two copies and that the distances between these clusters are ∼equal in the range of 0–4 copies (see Zmienko et al., 2016 for further discussion on the distances between the clusters in MLPA assays).

### Validation of the Results

Regardless of the number of probes and samples used, we recommend to verify the positive MLPA results with an independent technique. We advise performing droplet digital PCR (ddPCR) on selected samples, as this approach allows for estimating gene copy numbers at the same or even higher range, as the MLPA procedure described in this protocol (Zmienko et al., 2016). Additionally, ddPCR generates amplicons of ∼60–200 bp, therefore allows for genome assaying at similar resolution as MLPA.

## Conclusion

In this work, we described the protocol for the simple MLPA-based CNV genotyping in plants, with particular emphasis on the model plant *Arabidopsis*. We provided a description of the probe design process, experimental setup, and data analysis. We also discussed the results of the exemplar multiplex assay and showed that the MLPA method is very robust and is a rich source of information regarding the CNV in the analyzed samples. The abundant genomic data obtained for a growing number of species as a part of large-scale sequencing projects, highlight CNV as the major contributor to natural diversity at a genotype level (Zarrei et al., 2015; 1001 Genomes Consortium, 2016; Bai et al., 2016). Gene duplication has been considered the major factor driving long-term evolution and gene birth by sub- and neofunctionalization of the duplicated copies (Conant et al., 2014). Some regions in the genome may be more prone to CNV than the others, due to their specific structural features, that will locally induce the mechanisms leading to CNV formation, e.g., non-allelic recombination (Zmienko et al., 2016). The duplication / deletion events may have also consequences on organism’s fitness and contribute to the adaptation to environmental challenges, as well as to coevolutionary interactions between host and pathogen or a symbiont (reviewed in: Kondrashov, 2012, Żmieńko et al., 2014). Remarkably, the protein coding genes displaying CNVs are often related to environmental stress response and pathogen resistance (Cook et al., 2012; Maron et al., 2013). The creation of high-confidence CNV maps and assessing the gene copy number in large populations will enhance the studies on the evolution of genomes in the context of CNV origin, fixation and the impact on the phenotype. These data can be later combined with the results of the transcriptomic, proteomic, metabolomics, protein interaction, phenotyping, and other studies). We recently used the MLPA method to genotype *MSH2, AT3G18530*, and *AT3G18535* copy number in a set of 189 natural accessions. Based on these results, we were subsequently able to reveal the recurrent nature of *AT3G18530* and *AT3G18535* duplications/deletions and to dissect the structural features that promoted non-allelic homologous recombination, leading to a widespread occurrence of the *AT3G18530* and *AT3G18535* genes deletion in nature (Zmienko et al., 2016).

This protocol will enable potential users to introduce the MLPA technique in plant genetic and population biology studies. The technique is multiplexable and very well suited for verification of WGS-based analyses or for rapid characterization of copy number status across a region of interest in large populations. Notably, once designed, the individual MLPA probes may be used in various combinations according to one’s needs, providing that the lengths of the probes in one assay are unique. We believe that the MLPA protocol presented in the current work will contribute to accelerating the discovery of new associations between CNV and important traits in plants.

## Author Contributions

AS-C prepared DNA samples, performed MLPA assays, analyzed data, helped prepare figures, and draft the manuscript. MM-Z performed template optimization experiments. MM-S helped design the MLPA probes and set up the assay. PK analyzed the data and helped draft the manuscript. MF contributed to the conception of the work and revised the manuscript. AZ conceived of and designed the study, analyzed data, oversaw the research, prepared figures, and wrote the manuscript. All authors read and approved the final manuscript.

## Conflict of Interest Statement

The authors declare that the research was conducted in the absence of any commercial or financial relationships that could be construed as a potential conflict of interest.

## Footnotes

1 https://gbrowse.arabidopsis.org/cgi-bin/gb2/gbrowse/arabidopsis/

2 http://tools.1001genomes.org

3 http://www.mrc-holland.com/

4 http://arabidopsis.info/

5 http://1001genomes.org

6 http://www.mlpa.com/elearning/tswizard/
