# Supplementary material for: MLPA-Based Analysis of Copy Number Variation in Plant Populations
Source: Front Plant Sci. 2017 Feb 21;8:222. doi: 10.3389/fpls.2017.00222 (PMC5318451; doi:10.3389/fpls.2017.00222)
Supplement: Supplementary file 2 [file Presentation_1.PDF]

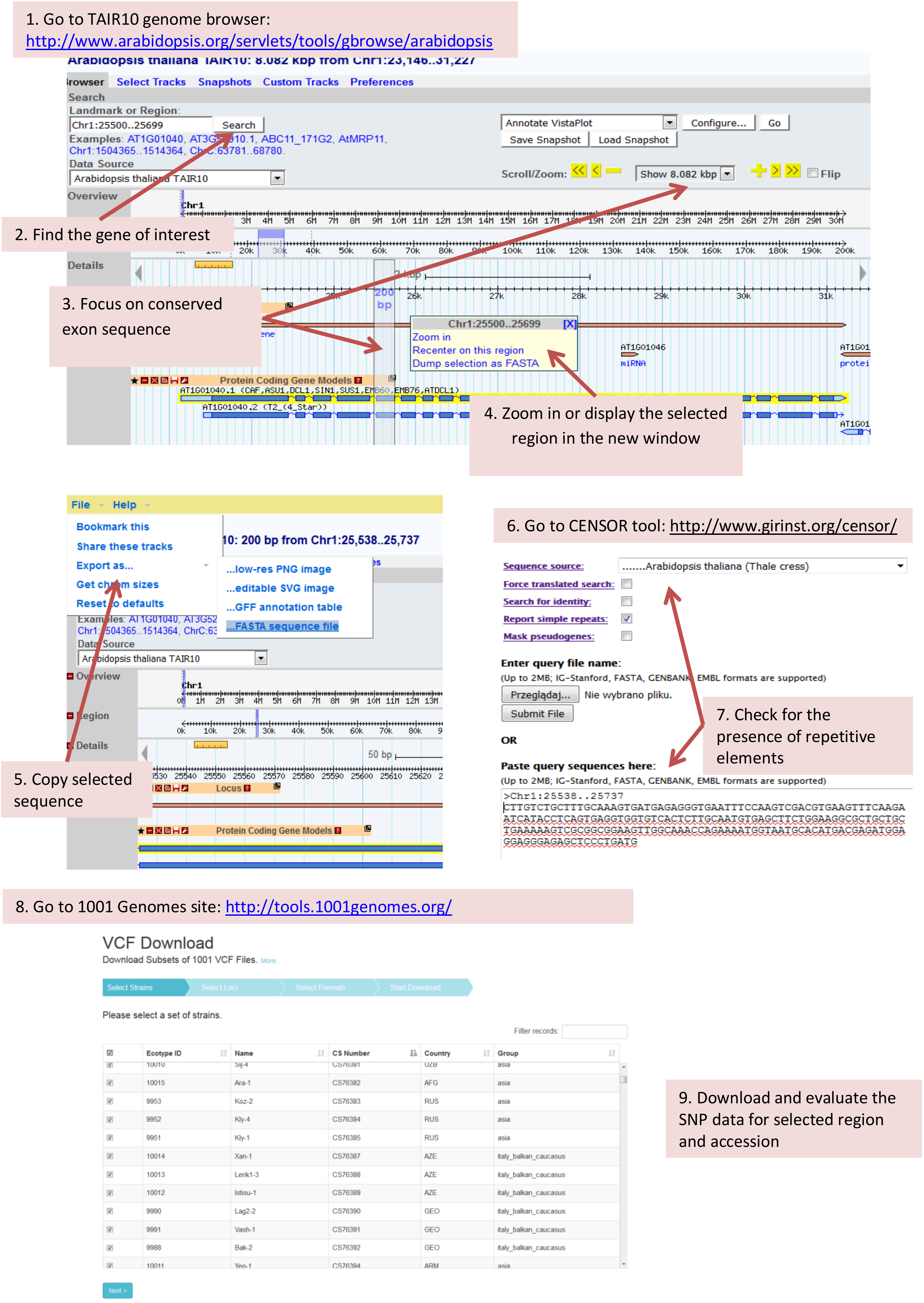

Supplementary Figure S1. The schematic procedure of the MLPA half-probes design

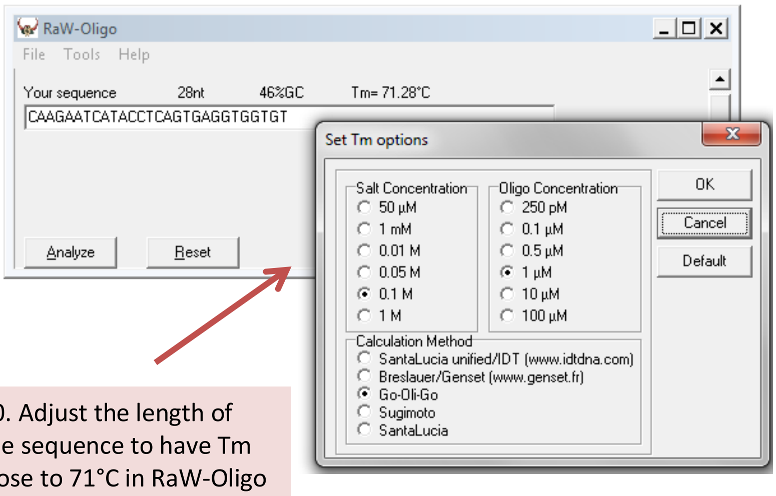

10. Adjust the length of the sequence to have Tm close to 71°C in RaW-Oligo

11. Go to: http://blast.ncbi.nlm.nih.gov/Blast.cgi Select Nucleotide BLAST

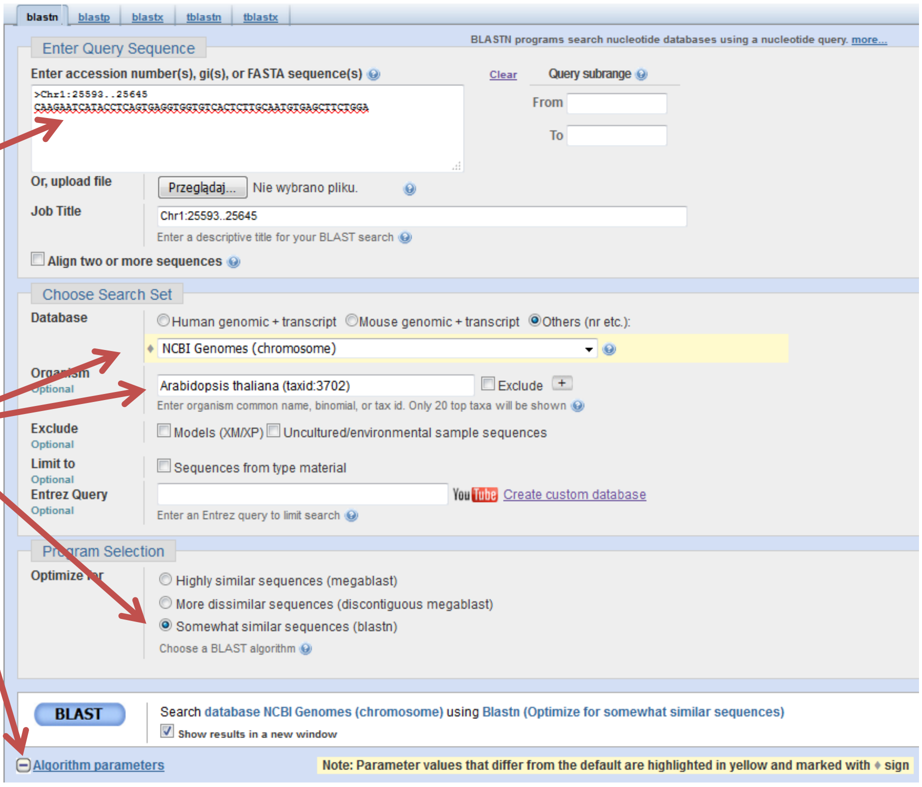

12. Paste the joined sequence of paired TSSs

13. Select the parameters

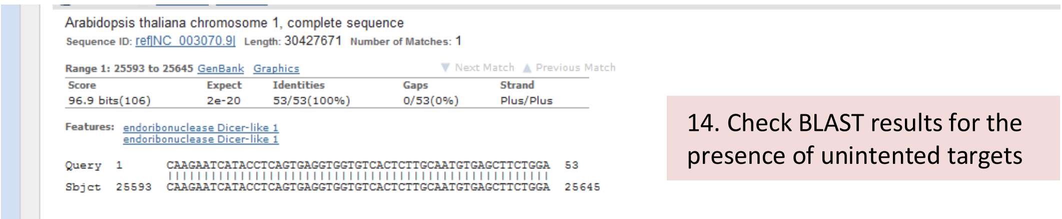

14. Check BLAST results for the presence of unintented targets

15. Use the MS Excel template to add primer and stuffer sequence and design the final half-probes

Left TSS	Tm of Left TSS	Range of Acceptable Left TSS Lengths	Left TSS Length	Left TSS Length Correct?	Left Half-Probe Sequence	Left Half-Probe Length	Anticipated Left Half-Probe Length	Left Half-Probe Correct?
		21-26	0	Not Used	Not Used	0	45	Not Used
		21-27	0	Not Used	Not Used	0	46	Not Used
CAAGAATCATACCTCAGTGAGGTGGTGT	71,28	21-29	28	Yes	GGGTTCCCTAAGGGTTGGACCAAGAAT	48	48	Yes
		21-30	0	Not Used	Not Used	0	49	Not Used
		21-32	0	Not Used	Not Used	0	51	Not Used

Supplementary Figure S1 (Continued).
