# Supplementary material for: MLPA-Based Analysis of Copy Number Variation in Plant Populations
Source: Front Plant Sci. 2017 Feb 21;8:222. doi: 10.3389/fpls.2017.00222 (PMC5318451; doi:10.3389/fpls.2017.00222)
Supplement: Supplementary file 3 [file Presentation_2.PDF]

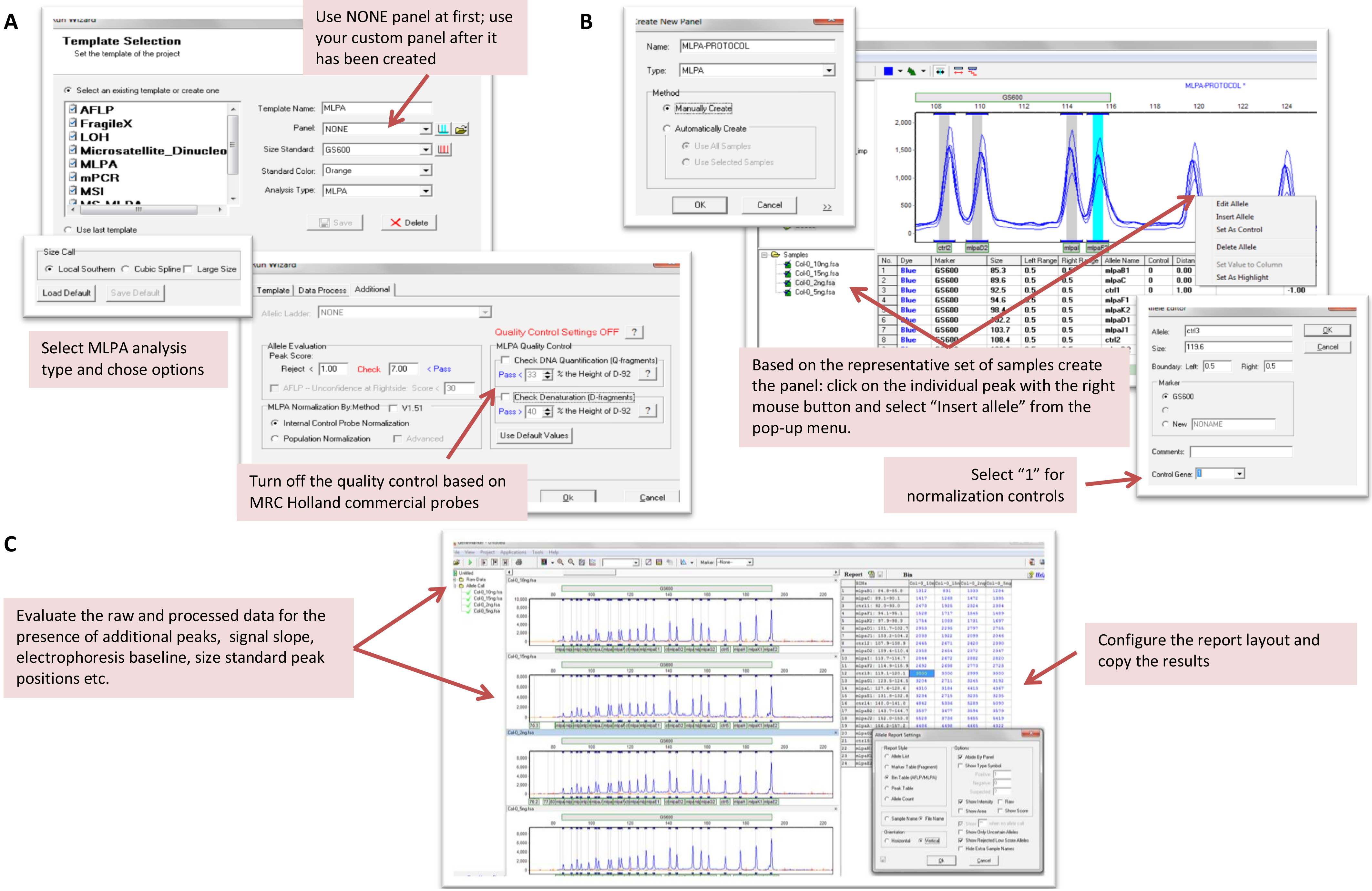

Supplementary Figure S2. Analysis of MLPA electropherograms in GeneMarker. A. Run analysis options; B. Manual creation of the MLPA panel ; C. Data evaluation and creation of the intensity report.
