# Supplementary material for: MLPA-Based Analysis of Copy Number Variation in Plant Populations
Source: Front Plant Sci. 2017 Feb 21;8:222. doi: 10.3389/fpls.2017.00222 (PMC5318451; doi:10.3389/fpls.2017.00222)

Supplementary Figure S3. Inappropriate peak recognition by GeneMarker software may lead to false „no call" result.

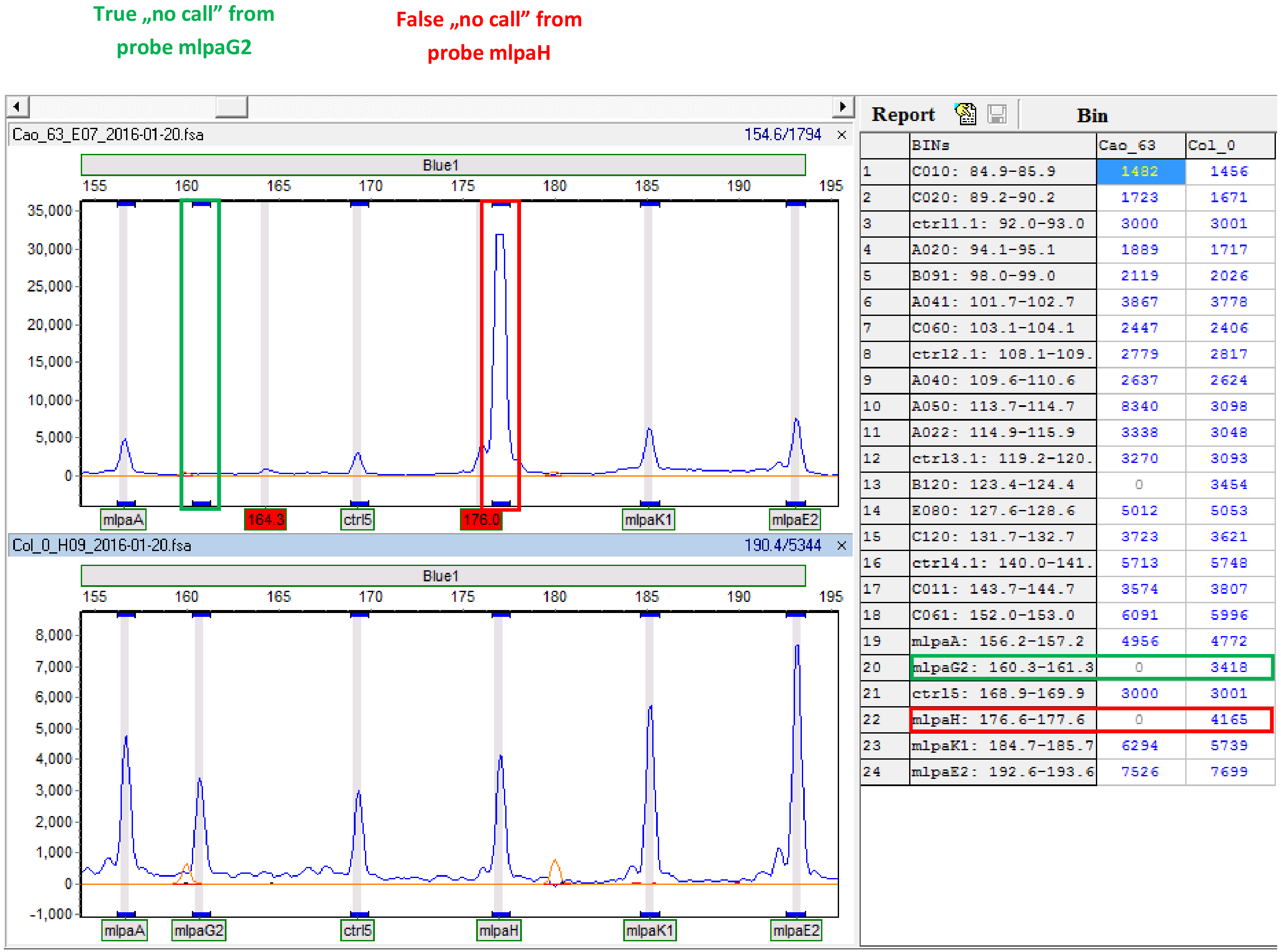

Supplement: Supplementary file 4 [file Presentation_3.PDF]
